# School's out: what are urban children doing? The Summer Activity Study of Somerville Youth (SASSY)

**DOI:** 10.1186/1471-2431-10-16

**Published:** 2010-03-24

**Authors:** Alison Tovar, Keith Lividini, Christina D Economos, Sara Folta, Jeanne Goldberg, Aviva Must

**Affiliations:** 1 John Hancock Research Center on Physical Activity, Nutrition and Obesity Prevention, Gerald J and Dorothy R Friedman School of Nutrition Science and Policy, Tufts University, 150 Harrison Ave, Boston, MA 0211, USA; 2 Department of Public Health and Community Medicine, Tufts University, 136 Harrison Avenue, Boston, MA 02111, USA

## Abstract

**Background:**

Research indicates that in the United States, children experience healthier BMI and fitness levels during school vs. summer, but research is limited. The primary goal of this pilot study was to assess where children spend their time during the months that school is not in session and to learn about the different types of activities they engage in within different care settings. A secondary goal of this pilot study was to learn what children eat during the summer months.

**Methods:**

A nine-week summer study of 57 parents of second and third grade students was conducted in an economically, racial/ethnically and linguistically diverse US urban city. Weekly telephone interviews queried time and activities spent on/in 1) the main caregiver's care 2) someone else's care 3) vacation 4) and camp. Activities were categorised as sedentary, light, moderate, or vigorous (0-3 scale). For each child, a mean activity level was calculated and weighted for proportion of time spent in each care situation, yielding a weighted activity index. On the last phone call, parents answered questions about their child's diet over the summer. Two post-study focus groups were conducted to help interpret findings from the weekly activity interviews.

**Results:**

The mean activity index was 1.05 ± 0.32 and differed between gender (p = 0.07), education (p = 0.08) and primary language spoken in the household (p = 0.01). Children who spent a greater percentage of time in parent care had on average a lower activity index (β = -0.004, p = 0.01) while children who spent a greater percentage of time in camp had a higher activity index (β = 0.004, p = 0.03). When stratified into type of camp, percentage of time spent in active camp was also positively associated with mean activity index (β = 0.005, p =< 0.001). With regards to diet, after adjusting for maternal education, children who attended less than five weeks of camp were four times more likely to eat their meals in front of the TV often/almost all of the time (OR = 4.0, 95%CI 1.0-16.2, p < 0.06).

**Conclusions:**

Summer activities and some dietary behaviours are influenced by situation of care and socio-demographic characteristics. In particular, children who spend a greater proportion of time in structured environments appear to be more active. We believe that this pilot study is an important first step in our understanding of what children do during the summer months.

## Background

The dramatic increase in the prevalence rates for overweight and obesity in children over the past several decades is a particular public health concern among low-income communities [1] and among ethnic minorities [2]. In urban environments the relative lack of recreational facilities and green space, difficulty in promoting walkability and bikeability, and concern over neighbourhood safety may limit outdoor activity, while lack of full-service supermarkets may limit access to healthful food products [3-7]. Overall, this environment can lead to decreased physical activity and increased intake of energy dense foods. Recent studies have shown that rate of change in BMI over the summer is larger than during the school year [8-10]. This has been shown to be true especially for black and Hispanic children and for children who are already overweight [8]. The reason for this pattern has yet to be firmly established. The popular press has speculated [11,12] that both diet and physical activity play a role in weight increases during the summer.

Some evidence suggests that physical fitness declines during the summer months [13,14]. In one study of 17 children, fitness levels decreased, while insulin levels and body fat percentage increased during the summer break [14]. A second study, which included 178 elementary school children, found significant physical fitness improvements during the school year but not during summer recess [13]. Contrary to these findings, a few studies have shown that children are more active during the summer months compared to the winter [15-17]. These studies however, used different activity methods, such as pedometers, and were completed in European populations, which have school and holiday calendars that differ from those in the United States. The majority of these studies point to an increase in overweight and obesity as well as a decrease in physical fitness during the summer months.

Energy dense foods, large portion sizes and low intake of fruits and vegetables have been identified as contributors to the increasing rates of obesity among children [18]. A number of studies suggest that school plays a role in promoting a more healthful diet [19,20]. School policies have been effective in improving the food environment and dietary intake in schools [21]. A review of prevention interventions suggests that combined diet and physical activity school-based interventions may help prevent children from becoming overweight [22]. One recent study, completed among a nationally representative sample of 2,314 children, found that on a typical school day, children consumed 527 "empty calories" during a 24-hour period and that eating at home provided the highest mean energy from low-nutrient, energy dense foods (276 kcal at home vs. 174 kcal at school and 78 kcal at other locations) [20]. No studies have looked at children's dietary intake specifically during the summer months when children are not at school.

Although there is emerging evidence that children gain more weight than they might be expected to for normal growth over the summer, no studies, to our knowledge, have explored the possible contributory behavioural factors. Therefore, the primary goal of this pilot study was to assess where children spend their time during the months that school is not in session and to learn about the different types of activities they engage in within different care settings. A secondary goal of this pilot study was to learn what children eat during the summer months.

## Methods

### Study Setting

This study was conducted in Somerville, Massachusetts, a densely populated city with more than 15,000 people per square mile and a total population of 77,000 [23]. This ethnically, racially, and culturally diverse city serves as the point of entry for many new immigrants, with over 50 different languages spoken. Most children attend public schools (more than 80%) [24] and 60% participate in the free and reduced-price school lunch program. This locale was selected for this pilot study because it represents a typical diverse, urban community, and because of the strong relationship between the Somerville community and Tufts University that was established during the "Shape Up Somerville" study [25].

### Study design

#### Recruitment

Primary caregivers of second and third grade children were recruited through a bilingual (English-Spanish) flyer sent home via backpack to parents of the 795 children in all second and third grade classrooms in Somerville Public Elementary Schools (7 elementary schools). No attempt was made to reach any student who was not present at school on the day flyers were distributed. The flyer included a brief explanation of the study, information on the study stipend, and contact information for parents interested in enrolment. Five trained interviewers (one bilingual in Spanish) received initial calls from interested parents who responded to the flyer. Our goal was to recruit parents until we reached our target sample of 58, given that this was a pilot study. Our target of 58 was based on resource constraints and an a priori assumption that 55-60 subjects would provide a reasonable picture of the pattern of activities for urban childrenin this age group. This first phone contact served as an enrollment call. During the call the interviewer described the study, outlined the parent's/guardian's participation, reviewed the voluntary nature of the study and confidentiality safeguards, and screened for eligibility (resident of Somerville, not going to be away on vacation for more than 2 weeks during the summer, English or Spanish language spoken, and access to a telephone). If parents were eligible and interested, the interviewer collected basic demographic information, including number of children in the household, level of parental education, race/ethnicity and employment status. This pilot study was approved by the Tufts University Institutional Review Board (IRB).

After the enrollment call, data were collected from parents over the phone using a short structured interview once each week on a pre-determined day (generally Friday and Saturday) and time for nine consecutive weeks between the end of June and end of August. The structured interview included questions on what the child did during the daytime hours on weekdays, including the types of activities the child engaged in. We chose to study only the daytime hours because we wanted to understand the types of summer activities that replace school and after-school activities. The interview was structured into four different care situations: 1) time spent on vacation; 2) time spent in summer camp (camp care); 3) time spent under someone else's care including siblings, other family members, nanny/babysitters and neighbours (other care); and 4) time spent under the main caregiver's care (parent care). For each of these situations, parents were asked what types of activities their children performed, on which days of the week, and how much time they spent doing these activities. Parents were also asked to rate how much time their child spent indoors for each of the situations, on a scale from 1-5, ranging from "almost all or all of the time" to "almost none or none of the time" for each week. The same script was used each week. The interviewers recorded all of the information on paper forms.

On the penultimate call, parents were asked if they were interested in answering additional questions about their child's diet over the summer during their last call. The dietary questionnaire used was slightly modified from the validated Youth-Adolescent Questionnaire [26] in order to capture the summer months and to make it age-appropriate. This instrument included 32 items that asked about dietary behaviours, and specific beverage, fast food, and fruit and vegetable consumption (instrument available from AT upon request).

During the last call parents were also invited to participate in a post-study focus group, which was designed to help interpret some of the findings from the weekly activity interviews. Interested parents were subsequently contacted by a research assistant and given a time and date to attend one of two focus groups. Focus groups were held within the community at central locations and participants received a $50 gift card for participation. Both groups lasted approximately one hour, and were audio-taped and transcribed. Two reviewers identified concepts and themes in the narratives by review of the transcripts.

### Data Analysis

Interview data were entered into the survey forms using Survey Monkey (SurveyMonkey.com, Copyright ^©^1999-2009) set up with range and logic checks. Weekly data were extracted into MS Excel, and open-ended questions coded into categories. Data were analysed using SAS (version 9.0).

We examined physical activity in three different care situations: parent care, other care (care provided by other family members or paid childcare provider), and camp care. Camp care was further categorised to reflect the different types of summer camps attended as sports (e.g. soccer camp), art (e.g. music or theatre camp), academic (e.g. reading camp), and traditional day camps (e.g. YMCA, Boys and Girls Club). Activities engaged in while on vacation were not included. Activities reported by parents in the different care situations were coded into four categories, (0-3), as sedentary, light, moderate or vigorous to reflect activity intensity, using Ainsworth's adult and youth compendia as guides [27,28]. Finally, to categorise children who were in more structured environments vs. those who were not, we defined "structured summer" as attendance at an active camp for a minimum of at least three days a week for three or more weeks. This cut-off point provided groups of comparable size.

A mean activity level was calculated for each week and care situation (parent care, other care and camp care) for each child. In order to make comparisons among children, the mean activity level was then weighted for the proportion of time spent in the different care situations. For each week, this proportion of time spent in a given situation was derived by dividing the total number of hours spent in each of the situations by the total number of weekly hours reported. The mean weekly care-specific activity level was then multiplied by the proportion of time spent in each situation and the values for each situation were summed. These values were then averaged across the weeks to derive an overall activity level for each child; we called this measure the subject's activity index. The activity index has no units and a theoretical minimum of 0 (a child who is sedentary all the time, in all situations) and a maximum of three (a child who is active all the time, in all situations). There were six parents who did not report hours spent in any situation on one of the nine weeks, these weeks were removed from the final analysis. Demographic information was summarised and reported as percentages. For descriptive analysis, we utilised t-tests to compare overall means for continuous variables and χ^2 ^to test for differences in proportions for categorical variables. A paired t-test was used to compare activity indices for the same child between weeks spent in camp and weeks not spent in camp (where weeks spent in camp is an average of at least three weeks). To examine the association between the activity index and care situation multivariable regression was performed. The final models accounted for the potential influence of demographic characteristics which were significant in the t-tests (child gender, mother's level of education, and whether a language other than English was spoken in the household).

Logistic regression was used to model the relation between camp attendance and specific dietary behaviours. Camp attendance was stratified at 5 weeks of camp attendance, the median value among attendees. This cut-off point provided groups of comparable size. Because of the exploratory nature of this study, we set our alpha level at 0.10.

## Results

A total of 58 parents were recruited. One subject was excluded after enrolment due to language/communication problems. Overall, 93% of interview calls were completed over the 9 weeks of summer. Among the 57 participating families, 67.0% of the children were male, 58.6% were white, 15.5% were Asian/Pacific Islanders, 12.1% were Hispanic and 3.5% were Black. 32.8% reported speaking a language at home other than English (Table 1). Mothers completed the majority of the calls (87.9%) and more than half of the respondents (54.6%) reported working during the summer. Of those who were employed outside the home, 61.5% reported working full time (Table 1).

**Table 1:** Demographic characteristics of participants

(n = 57)	**No**.	%
Gender		
Male	39	67.0
Race		
Hispanic	7	12.1
White	34	58.6
Black	2	3.5
Asian Pacific Islander	9	15.5
Multiracial	1	1.7
Other	5	8.6
Education level of mother		
Less than high school	6	10.3
High school graduate	14	24.1
Some college	10	17.2
College graduate	11	19.0
Graduate Degree	17	29.3
Other language besides English spoken in household		
Yes	19	32.8
No	39	67.2
Number of other children in household		
0	11	19.0
1	31	53.4
2	12	20.7
3	3	5.2
4	1	1.7
Number of adults in household		
1	5	8.6
2	47	81.0
3	3	5.2
4	3	5.2
Relationship to child of person completing calls		
Mother	51	87.9
Father	6	10.3
Guardian	1	1.7
Working during Summer		
Yes	30	54.6
No	25	45.5
Full Time	16	61.5
Part Time	10	38.4

*Sample size varies due to missing data

Complex care arrangements were frequent for children in the study, with multiple caregivers and attendance at more than one kind of camp common. Approximately 98.0% of parents reported that their children spent some time under parent care, 74.0% reported that their child spent time under someone else's care and 77.0% reported that their child attended camp at some time during the summer. For children under the care of others, 56.0% were in the care of a non-parent adult family member, 25.0% in the care of a neighbour or friend, 14.0% in the care of a sibling, and 12.0% in the care of a nanny or babysitter at least one time during the summer. Twenty children (35.0%) attended sports camp and spent an average of 10 days going to this type of camp. Similarly, for the 11 children (19.0%) that reported attending art camps, they spent an average of 10 days attending. Twenty-six children (46.0%) reported attending academic camps and spent an average of 11 days at this camp. Twenty (35.0%) attended traditional day camps and spent an average of 16 days of the summer in this type of camp. With regards to time spent indoors, 60.0% reported that their child spent almost all or most of the time indoors during summer camp, 43.0% reported that their child spent almost all or most of the time indoors under someone else's care, and 67.0% reported that their child spent all or most of time indoors under parent's care.

### Activity

Children engaged in a range of activities of varying intensities. Typical sedentary activities included screen time (TV, computers or video games); arts and crafts; board games; travelling; and reading/writing. Light activities included walking, shopping, running errands, doing household chores, and playing with toys (i.e. Lego's). Typical moderate level activities included playing outside and swimming and vigorous activities included playing/training in sports such as basketball, baseball, hockey, football, karate, gymnastics and riding bikes.

Overall the mean (SD) activity index was 1.05 ± 0.32, with median of 1.0, and an inter-quartile range of 0.41. The mean activity index did not differ by parent employment status, child's race, number of adults in the household, or number of children in the household (p > 0.10) (Table 2). However, mean activity index differed between those households where another language besides English was spoken and households where only English was spoken (p = 0.01). On average, the mean activity index for English-only households was 0.21 units higher compared to those where another language was spoken. A similar trend was observed for gender and education. On average, the mean activity index for males was 0.16 units higher than females (p = 0.07). On average, the mean activity index for children whose mother had at least some college was 0.15 units higher than those who had less than a college degree (p = 0.08).

**Table 2:** Differences in mean activity index by demographic variable

(n = 57)	mean	std	*p-value*
Gender			
Male	1.11	0.30	
Female	0.95	0.33	0.07
Parent working			
Yes	1.00	0.29	0.14
No	1.13	0.34	
Education level of mother			
Less than college	0.95	0.36	0.08
At least some college	1.10	0.29	
Race			
White	1.10	0.29	0.10
Other	0.97	0.34	
Other language besides English spoken in household			
Yes	0.89	0.26	0.01
No	1.10	0.32	
Adults in the household			
Less than 2	1.18	0.32	0.36
2 or more	1.04	0.32	
Other children in the household			
Less than 2	1.04	0.32	0.76
2 or more	1.07	0.34	

**Figure 1:**
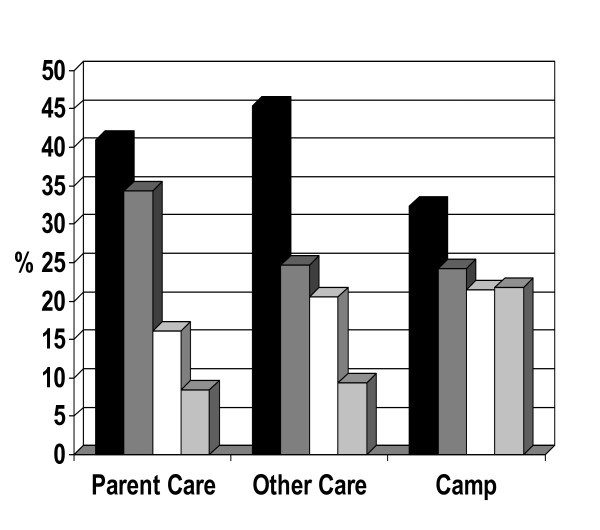
**Activity intensity by care situation**. Black = Sedentary, Dark Grey = Light, White = Moderate, Light Grey = Vigorous

Of the total number of activities reported throughout the summer, 39.0% were sedentary, 31.0% were light, 19.0% were moderate and only 12.0% were vigorous. When examined by care situation, 41.0% of the total number of activities were sedentary when under the care of their parents, 46.0% were sedentary when under the care of others, and while in summer camp, only 32.0% of the total number of activities children engaged in were considered sedentary (Figure 1). On average, children who attended an active camp for three weeks or more were significantly more active that those who attended for less than three weeks (p < 0.001). In a within-child comparison of camp vs. non-camp activity level, children's mean activity index was 0.35 units higher for weeks spent in active camps vs. weeks not spent in active camps (p < 0.01.) In a similar comparison between weeks spent in all types of camps vs. weeks not spent in camp, children's mean activity index was 0.17 units higher for weeks spent in camp vs. weeks not spent in camp (p = 0.01).

In separate models by care situation, we found that percentage of time spent in parent care was negatively associated with mean activity index in both unadjusted and adjusted analysis (β = -0.004, p = 0.01) while percentage of time spent in camp was positively associated with mean activity index (β = 0.004, p = 0.03) (Table 3). Percentage of time spent in other care was not significantly associated with mean activity index in either unadjusted or adjusted models. When stratified by type of camp, percentage of time spent in active camp was also positively associated with mean activity index (β = 0.005 p < 0.001). The opposite was true for non-active camps, where percentage of time spent in non-active camp was associated with lower activity index (β = -0.006, p = 0.04). In a final model that included other care, active camp and non-active camp, adjusting for gender, education and language, active camp was the only care situation which was significantly related to activity index (β = 0.005, p = 0.001).

**Table 3:** Linear regression analysis of activity index and situation

	Weighted Activity Level						
**(n = 57)**	**Unadjusted Model**			**Adjusted Model**			**R^2^**

	**β**	**SE**	***p-value***	**β**	**SE**	***p-value***	
Percent of time spent in parent care	-0.004	0.002	0.010	-0.004	0.002	0.008	0.27
Male				-0.102	0.083	0.227	
Education level of mother				0.090	0.080	0.273	
Primarily English spoken in household				0.181	0.084	0.035	

Percent of time spent in camp	0.004	0.002	0.007	0.004	0.002	0.026	0.24
Male				-0.064	0.087	0.460	
Education level of mother				0.045	0.088	0.610	
Primarily English spoken in household				0.198	0.085	0.023	

Percent of time spent in active camp	0.006	0.001	<.001	0.005	0.001	<.001	0.34
Male				-0.049	0.080	0.548	
Education level of mother				0.084	0.080	0.310	
Primarily English spoken in household				0.160	0.080	0.051	

Percent of time spent in non-active camp	-0.005	0.003	0.083	-0.006	0.003	0.042	0.23
Male				-0.110	0.086	0.206	
Education level of mother				0.141	0.092	0.131	
Primarily English spoken in household				0.155	0.088	0.084	

### Diet

A total of 54 (94.0%) parents completed the diet questionnaire to reflect usual dietary habits over the summer. Overall, 79.0% of children ate breakfast every day and 18.0% did so on most days during the summer. During a typical summer week, 38.0% of children ate lunch prepared away from home three or more times, but only 7.0% ate dinner prepared away from home. One quarter (25.0%) of parents reported that they ate three or fewer dinner meals together as a family either at home or outside of the home during a typical week in the summer. Approximately 24.0% of children ate three or more servings of fruit and only 13.5% ate three or more servings of vegetables per day during the summer months (Table 4). In contrast, more than 40.0% of parents reported that children ate chips and ice cream more than three times a week and approximately 30.0% ate baked goods more than three times a week. With regards to what children drank during the summer, 17.0% of parents reported that their child drank soda more than three times per week and 32.0% reported that their child drank sugar sweetened beverages (e.g. Gatorade, Kool-aid etc) more than three times per week. Over 63.0% reported that they drank 100% fruit juice and milk more than three times per week. Approximately 65.0% of the children consumed 2% or whole milk and 34.0% had flavoured milk (chocolate, strawberry of vanilla).

**Table 4:** Dietary characteristics of 54 children completing the diet questionnaire

	**No**.	%
Fruit consumption		
Never	2	3.7
1-2 times per day	39	72.2
3 or more times per day	13	24.0
Vegetable consumption		
Never	7	13.5
1-2 times per day	38	73.1
3 or more times per day	7	13.5
Soda consumption		
Never	28	52.8
1-2 times per week	16	30.2
3 or more times per week	9	17.0
Juice consumption (100%)		
Never	10	18.5
1-2 times per week	10	18.5
3 or more times per week	34	63.0
Sugar sweetened beverage consumption		
Never	13	24.1
1-2 times per week	24	44.4
3 or more times per week	17	31.5
Milk consumption		
Never	6	11.1
1-2 times per week	5	9.3
3 or more times per week	43	79.6
French Fries		
Never	20	18.5
1-2 times per week	25	46.3
3 or more times per week	9	16.7
Snack Chip consumption		
Never	10	18.5
1-2 times per week	22	40.7
3 or more times per week	22	40.7
Baked goods		
Never	5	9.4
1-2 times per week	33	62.3
3 or more times per week	15	28.3
Ice cream		
Never	1	1.9
1-2 times per week	22	40.7
3 or more times per week	31	57.4
Eat snacks away for home		
Never	9	17.0
1-2 times per week	20	37.7
3 or more times per week	24	45.2
Eat dinner away from home		
Never	22	40.7
1-2 times per week	28	52.0
3 or more times per week	4	7.4

Children who spent more time in camp had different dietary behaviours compared to those who attended less camp. In unadjusted analysis, children who attended fewer than five weeks of camp were 4.7 times more likely to have breakfast only a few days or never during a typical week in the summer (OR = 4.7, 95% CI 0.81-28.0 p = 0.08), and were 4.5 times more likely to eat meals in a room with the TV turned on often/almost all of the time (OR = 4.5 95% CI 1.2-17.6 p = 0.03) compared to those who attended more than five weeks of camp. After adjusting for maternal education, children who attended fewer than five weeks of camp were four times more likely to eat meals in a room with the TV turned on often/almost all of the time (OR = 4.0, 95%CI 1.0-16.2, p = 0.06); this association was marginally significant.

A total of 15 parents (26.0%) participated in two post-study focus groups. Parents' perceptions of how time was spent during the summer differed by their employment status. Those who were working described the transition from prescribed school activities to prescribed summer activities as almost seamless. Parents who did not work during the summer or who had flexible work schedules tended to see lower levels of structure during the summer as a relief from the time constraints of the school year and enjoyed the slower pace. Some of these parents said, however, that it was good that summer recess was limited because the kids began to get bored. There was a generally consistent feeling that summer rules regarding TV, bedtime hours, and snacks, particularly ice cream, were more relaxed in the summer. Parents perceived that children were more active overall during the summer than while they were in school, but observed that there were more organized activities and team sports during the school year.

## Discussion

The goal of this pilot study was to explore what children do during the summer months, where they spend time, what types of activities they engage in, and what they eat during the summer months. In this diverse sample, we found that children spend a great deal of time during the summer weeks under the care of their main caregivers followed by time in camp and someone else's care. Children in this sample spent a large proportion of time during the summer engaged in sedentary or light activities. We found that children who spend a greater proportion of time under parent care are less active on average, whereas those children who spend a greater proportion of time in camp are more active, after adjusting for potentially confounding factors. With regards to diet, children who attended less than five weeks of camp were more likely to eat meals in front of the TV more often compared to those that attended greater than five weeks of camp.

We found that children appear to be spending a large proportion of their time during the summer engaging in sedentary or light intensity activities and that the care environment appears to be associated with their level of activity. In this study, when children spend time in camp, a smaller proportion of the activities they perform are either sedentary or light activities. We also found that children who spend a greater proportion of time in camp, particularly active camp, have higher activity levels, after adjusting for possible confounding factors. Although studies on the types of activities that children engage in during the summer are limited, one physical education intervention study completed during the school months found that improvements in cardiovascular fitness achieved during the school year were lost during the summer break and that fitness levels decreased [14]. As suggested by Von Hippel et al. individuals may be more likely to gain weight when they are in a relatively unstructured environment[8]. Our data provide a possible mechanism that might underlie these changes, as children who spend a greater proportion of time under the care of their parent have lower average activity levels. Since a large percentage of the camps attended by children in the present study were either traditional day camps or sports camps, they may mimic the structured nature of physical education during the school months and might be expected to contribute to maintenance of cardiovascular fitness.

We also found that certain demographic characteristics such as gender, education and household language are associated with child activity levels. In our study, being female was associated with lower activity levels. Our results are similar to what others have found in this age group of children, for example NHANES III (1988-1994) analyses of 8-16 year old children found that 20.0% of US children participated in 2 or fewer bouts of vigorous activity per week, and the rate was higher in girls (26.0%) than in boys (17.0%) [29]. More recently Anderson et al. reported that female gender was associated with a higher probability of low active play and high screen time [30]. With regards to education which is often a proxy for socioeconomic status, previous studies have found that lower income populations are at higher risk for physical inactivity [31], explained in part by concern over neighbourhood safety and lack of outdoor activity [5]. Similarly, ethnic disparities with respect to physical activity in children have also been reported [32,33]. These socio-demographic characteristics may be markers for other factors that affect physical activity among children.

With regard to diet, we found that a large percentage of children are eating only one to two servings of fruits and vegetables per day during the summer months. This is consistent with national data from NHANES [34,35]. Nearly half of parents also reported that their children ate snack chips, ice cream and baked goods more than three times a week. Although we are unaware of studies that have examined diet during the summer, specifically these findings follow the national pattern of what children are eating. For example, mean intakes of fruit and vegetable are well below recommended amounts [35] and intakes of energy dense foods are high [36,37]. We also found that children who attend camp for more than five weeks during the summer are less likely to eat meals and snacks in front of the TV during the summer. This suggests that having a more structured environment may also provide benefits for children's diets. It is possible that children are having more screen time while consuming high-energy dense snack foods.

There are several limitations to this exploratory effort. First, to minimize the burden to subjects, the activity measures were crude, and the interview format limited our ability to quantify the exact time spent performing physical activity. Although our approach required us to make several simplifying assumptions for analysis, our primary goal was to explore what children were doing, where children were spending time, and what activities they were engaged in during the summer months. Also, parents in our pilot study were proxy reporters of their children's activities and the time children spent engaged in these activities. As proxy reporters, their reports are prone to error, particularly inasmuch as they may not be with their children and thus not fully aware of what happens during the day. However, several studies provide support for their use in observational studies of children [38-43]. Second, diet patterns were assessed using a modified food frequency questionnaire that had not been used previously to capture only summer months; thus its validity is uncertain. Third, our limited sample size may have precluded our observing statistically significant differences, although we set a liberal alpha level of 0.10 to avoid missing potentially meaningful effects. Finally, the amount of precipitation in Massachusetts in the summer of 2008 was atypically high; 20 out of the 45 days had some precipitation with a total summer rainfall of 9.4 inches, compared to an average of 8.0 inches [44]. During the weekly telephone interviews, parents often commented on the impact of the inclement weather. This could have affected activity levels during this time period, and further limits the generalizablity of the findings in this convenience sample.

## Conclusions

In conclusion, in this diverse urban sample, children's activities and some dietary behaviours during the summer are influenced by situation of care and certain socio-demographic factors. We believe that this pilot study represents an important early step in our understanding of what children do during the summer months when school is out. Future efforts would benefit from a larger sample size and more quantitative and/or objective assessment of physical activity in combination with self-reported measures. If these findings are confirmed, future interventions that evaluate more structured programs for all children during the summer may be warranted, and may ultimately contribute to moderating obesity in the paediatric population.

## Competing interests

The authors declare that they have no competing interests.

## Authors' contributions

All the authors contributed to the various stages of this study. AT contributed to the study design, supervised all the phases of the study, performed some of the statistical analysis, and drafted the manuscript. KL performed most of the statistical analysis and helped draft the manuscript. CE participated in the design of the study and revised manuscript. SF participated in the design of the study, collected focus group data and revised manuscript. JG participated in the design of the study and revised manuscript. AM conceived of the initial idea of the study, contributed to design of the study, revised the manuscript and contributed especially to the intellectual content. All the authors read and commented on the drafts and approved of the final version for submission.

## Pre-publication history

The pre-publication history for this paper can be accessed here:

http://www.biomedcentral.com/1471-2431/10/16/prepub
